# IL-4 Receptor-Alpha Signalling of Intestinal Epithelial Cells, Smooth Muscle Cells, and Macrophages Plays a Redundant Role in Oxazolone Colitis

**DOI:** 10.1155/2020/4361043

**Published:** 2020-01-17

**Authors:** Jennifer Claire Hoving, Roanne Keeton, Maxine A. Höft, Mumin Ozturk, Patricia Otieno-Odhiambo, Frank Brombacher

**Affiliations:** ^1^AFGrica Medical Mycology Research Unit, Institute of Infectious Diseases and Molecular Medicine, University of Cape Town, South Africa, MRC Centre for Medical Mycology at the University of Exeter, Geoffrey Pope Building Stocker Road, Exeter, UK; ^2^Institute of Infectious Diseases and Molecular Medicine (IDM), Department of Pathology, Faculty of Health Sciences, University of Cape Town, South Africa; ^3^International Centre for Genetic Engineering and Biotechnology (ICGEB), Cape Town Component, Cape Town, South Africa; ^4^South African Medical Research Council (SAMRC), South Africa

## Abstract

A hallmark of ulcerative colitis is the chronic colonic inflammation, which is the result of a dysregulated intestinal mucosal immune response. Epithelial barrier disruption which allows the entry of microorganisms eventually leads to more aggressive inflammation and potentially the removal of the colon. We have previously shown that the T helper- (Th-) type 2 cytokines, Interleukin- (IL-) 4 and IL-13, mediate CD4+ T cell- or B cell-driven inflammation in the oxazolone-induced mouse model of ulcerative colitis. In contrast, mice deficient in the shared receptor of IL-4 and IL-13, IL-4 receptor-alpha (IL-4R*α*), on all cells develop an exacerbated disease phenotype. This suggests that a regulatory role of IL-4R*α* is required to protect against severe colitis. However, the cell populations responsible for regulating the severity of disease onset through IL-4R*α* in colitis are yet to be identified. By deleting IL-4R*α* on specific cell subsets shown to play a role in mediating colitis, we determined their role in a loss of function approach. Our data demonstrated that the loss of IL-4R*α* signalling on intestinal epithelial cells, smooth muscle cells, and macrophages/neutrophils had no effect on alleviating the pathology associated with colitis. These results suggest that IL-4/IL-13 signalling through IL-4R*α* on nonhematopoietic intestinal epithelial or smooth muscle cells and hematopoietic macrophage/neutrophils has a redundant role in driving acute oxazolone colitis.

## 1. Introduction

Intricate regulatory mechanisms in the intestine maintain homeostasis with the dysregulation of this balance often resulting in devastating inflammatory bowel disease. Ulcerative colitis (UC) is an inflammatory bowel disease mediated by an atypical T helper- (Th-) type 2 immune response. While the focus of the mechanism of disease has been predominantly on NK T cells producing IL-13, the involvement of other Th2 cell types has also been implicated [1, 2]. In the oxazolone-induced colitis mouse model, Interleukin- (IL-) 13 is the main cytokine responsible for the pathology seen [1]. Based on both animal and patient data, the proposed mechanism for UC is that antigen is presented to and taken up by lamina propria antigen-presenting cells (APCs) including dendritic cells or macrophages. These APCs then present antigen to NK T cells which are activated to secrete IL-13. NK T cells potentially act directly on epithelial cells, but IL-13 production by these cells is suggested to be the primary cytokine mediating UC, as it causes changes in the epithelial cell barrier and activates other Th2 immune cells [3–6]. Furthermore, IL-13 is upregulated in ulcerative colitis patients and has been shown to increase colon epithelial permeability by inducing apoptosis [5, 6]. Our own studies have demonstrated that CD4+ T cells and B cells deficient in the IL-4/IL-13 common receptor IL-4R*α* are protected from oxazolone-induced colitis [2]. Based on our results, we concluded that CD4+ T helper- (Th-) type 2 cells producing IL-13 and B cells producing IgE were responsible for mediating colitis in mice [2]. While IL-4/IL-13 signalling on both T cells and B cells contributes to the disease phenotype, our previous work showed that IL-4R*α* deletion on all cell types significantly exacerbated disease compared to wild-type mice [7]. This suggests the role of a yet to be identified cell type in preventing disease through IL-4R*α* signalling. In an attempt to identify the responsible IL-4R*α*-expressing cell type, we expanded our studies to include other cells known to be involved in colitis, including intestinal epithelial cells, smooth muscle cells, and macrophages.

Disruption of the epithelial barrier in the intestine has been shown to contribute to the severity of disease, with IL-13 influencing epithelial cell function and driving apoptosis in these cells. Furthermore, IL-4R*α*-responsive smooth muscle cells have been shown to enhance the Th2 response in disease pathology. Therefore, we initially focused our studies on nonhematopoietic cells, such as intestinal epithelial cells or smooth muscle cells. Here, we used cre-loxP recombination to specifically delete IL-4R*α* from either intestinal epithelial cells (Villin^cre^IL-4R*α*^-/lox^) [8] or smooth muscle cells (SM-MHC^cre^IL-4R*α*^-/lox^) [9, 10]. Using this loss of function approach, we demonstrated that signalling of IL-4/IL-13 via the IL-4R*α* played a redundant role in both intestinal epithelial cells and smooth muscle cells. Although the disease pathology seemed to be slightly reduced in smooth muscle cell-deficient mice, this was not significantly different when compared to hemizygous littermate control mice.

While macrophages, one of the most abundant leucocytes in the intestinal mucosa, maintain gut homeostasis by discriminating harmful antigens, they are also responsible for the pathogenesis associated with inflammatory disease [11, 12]. Hence, there is a potential for novel therapeutic approaches, which may target macrophages specifically [12]. Macrophages can be proinflammatory and classically activated (M1) or anti-inflammatory and alternatively activated (M2). The latter is driven by the Th2 cytokines, IL-4 or IL-13, through the common IL-4R*α* [13]. While tissue repair through arginase production, helminth clearance, and protection against Th1-mediated colitis in a mouse model of Crohn's disease are some of the beneficial effects of M2 macrophages, they also mediate detrimental allergic responses in predisposed individuals [13]. Furthermore, it is accepted that the composition and functions of intestinal macrophages differ in the inflamed gut of UC and Crohn's disease patients [14]. In the current literature, much more is known about the role of both M1 and M2 macrophages in Crohn's disease, with little described about these cells in UC.

To address the role that IL-4/IL-13 signalling plays on macrophages in oxazolone-induced colitis, we used the macrophage/neutrophil-specific IL-4R*α*-deficient mouse strain (LysM^cre^IL-4R*α*^-/lox^) [15]. While the evidence is compelling that M2 macrophages could influence the outcome of disease, macrophage/neutrophil-specific IL-4R*α*-deficient mice maintained a disease phenotype comparable to hemizygous littermate controls. Our data suggests a redundant role for IL-4/IL-13 signalling on macrophages in acute oxazolone-induced colitis. Combined, our data suggests that IL-4/IL-13 signalling on intestinal epithelial cells, smooth muscle cells, macrophages, and neutrophils plays a redundant role in oxazolone colitis. Therefore, the IL-4R*α*-expressing cell type responsible for the regulation of IL-4/IL-13 signalling during oxazolone colitis remains to be determined.

## 2. Materials and Methods

### 2.1. Mice

Previously generated male Villin^cre^IL-4R*α*^-/lox^ [8], SM-MHC^cre^IL-4R*α*^-/lox^ [9, 10], LysM^cre^IL-4R*α*^-/lox^ [15], or hemizygous littermate control mice on a BALB/c background were used in the experiments. Villin^cre^, SM-MHC^cre^, or LysM^cre^ mice were crossed with IL-4R*α*^lox/lox^ BALB/c mice and complete IL-4R*α*^−/−^ BALB/c mice to generate hemizygous Villin^cre^IL-4R*α*^-/lox^ or SM-MHC^cre^IL-4R*α*^-/lox^ mice. Mice were backcrossed to a BALB/c background for 9 generations. Mice were genotyped as described previously. All mice were housed in specific pathogen-free conditions at the University of Cape Town, South Africa, and experiments were approved by the University's Animal Ethics Committee.

### 2.2. Peritoneal Lavage and Colon Cell Isolation

Cells were isolated from the peritoneal cavity of naïve mice by lavage using 10 ml IMDM/10% FCS and stained for multiparameter flow cytometry (FACS). Cells were isolated from colonic tissue using a previously described protocol, slightly modified [16]. Briefly, the colon was removed from 4 mice per group, flushed with PBS, and cut open longitudinally. After rinsing with DPBS, the colons were cut into 3-5 mm pieces, pooled from 4 individual mice. The intestinal layers were mechanically dissected, and the epithelial layer was discarded using 5 mM EDTA/10 mM HEPES/DPBS (free of Ca2+ and Mg2+) by shaking at 37°C for 15 min, repeated 3 times. The remaining lamina propria and muscle layer were digested with 0.5 mg/ml collagenase type VIII and 1 mg/ml DNase I in IMDM containing HEPES by shaking for 40-50 min at 37°C. After passing the cells through a 70 *μ*m sieve, the single cell suspension was layered over a Percoll gradient of 30% and 100%. The cell layer at the 30/100% interphase was collected, washed in IMDM/10% FCS, and stained for FACS.

### 2.3. Flow Cytometry

IL-4R*α* surface expression was detected on live cells isolated from the peritoneum or lamina propria by phycoerythrin (PE) anti-CD124 (IL-4R*α*, M-1). Cell subpopulations were identified with Alexa Fluor 700, BD Horizon V450, APC, or PE-Cy7 for F4/80, Ly6G, CD11c, and CD11b (BD Pharmingen). Stained cells were then acquired on a LSRII flow cytometer (BD Bioscience), and data were analyzed using FlowJo software (TreeStar Inc.).

### 2.4. Induction of Colitis by Haptenating Agent Oxazolone

Oxazolone-mediated colitis was induced in BALB/c mice as previously described [2, 7]. Essentially, mice anesthetized with ketamine (80 mg/kg) and xylazine (7.5 mg/kg) prepared in sterile PBS and monitored until fully conscious were sensitized on the shaved abdomen by application of 3% oxazolone (4-ethoxymethylene-2-phenyl-2-oxazolin-5-one; Sigma-Aldrich) in 100% ethanol (150 *μ*l) followed 7 days later by intrarectal administration of 1% oxazolone in 50% ethanol (150 *μ*l). Control mice were sensitized and challenged with ethanol only. All treated experimental and control animals were monitored and weighed daily. Once the animals showed signs of moderate discomfort or distress, they were weighed twice daily and monitored three times daily. Animals in severe distress or those that had lost ≥20% bodyweight were euthanized immediately using 5% halothane in air, and death was confirmed by cervical dislocation. Surviving mice were euthanized at 3 days postchallenge for immunopathological analyses.

### 2.5. Disease Activity Index

Oxazolone-treated BALB/c mice develop rapid onset colitis marked by weight loss and disease activity index (animal distress score). Disease progression was determined as previously described [2, 7] with weight loss measured as a percentage of starting weight and distress scored at day 2 postchallenge (Table 1 [2, 7, 17]). Colon length was measured from the anus to the caecum and recorded as an indication of inflammation.

### 2.6. Histological Assessment of Colitis

Colon sections taken 1 cm from the anus for colitis experiments were processed as previously described [2, 7] and stained with hematoxylin and eosin (H&E) for inflammatory cells or Periodic acid-Schiff (PAS) reagent for mucus-producing goblet cells. Semiquantitative histopathological grading of oxazolone-induced colitis was determined as previously described [7]. Mice were graded on 5 criteria: (1) presence of mononuclear cells, (2) reduced goblet cells, (3) epithelial injury, (4) granulocyte infiltration, and (5) edema. Each criterion was scored from 0 to 3, and the total score was added resulting in a total additive score between 0 (no colitis) and 15 (maximal colitis activity). The histological mucus index (HMI) was used to quantify colonic goblet cells in individual mice from PAS-stained sections as previously described [9]. Briefly, colon sections were photographed at a magnification of ×40 and overlaid with a standard grid. The total number of epithelial cells was divided by the number of mucus-positive squares to determine the HMI. Histology sections were processed and stained by the Department of Surgery, Groote Schuur Hospital.

### 2.7. Statistical Analysis

Values are given as the mean ± SEM, and significant differences were determined using unpaired two-tailed Students *t*-test or one-way ANOVA using a Bonferroni posttest for multiple comparison (GraphPad Prism). Values of *p* < 0.05 were considered significant.

### 2.8. Ethical Considerations

All animal experiments described were performed by trained and South African Veterinary Council registered researchers. The study was approved by the University of Cape Town Animal Ethics Committee (AEC) (012/025), and all methods were performed in accordance with the guidelines and regulations of the AEC.

## 3. Results

### 3.1. IL-4R*α* Signalling on Intestinal Epithelial Cells Plays a Redundant Role in Oxazolone-Induced Colitis

**Figure 1:**
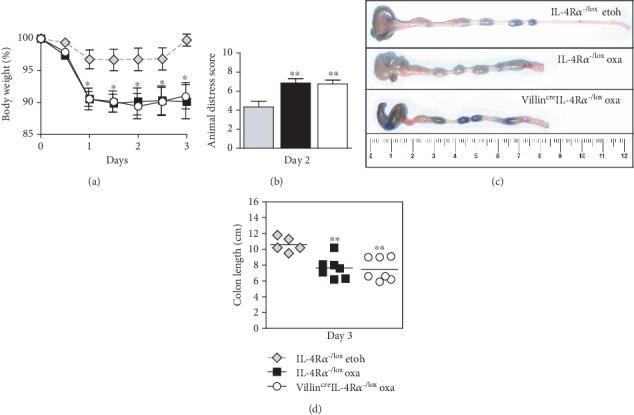
Oxazolone colitis develops in intestinal epithelial cell-specific IL-4R*α*-deficient mice. Villin^cre^IL-4R*α*^-/lox^ mice were not protected from the onset of oxazolone-induced colitis compared with littermate control mice, shown by comparable (a) weight loss as a percentage of starting weight and (b) increased distress (day 2). (c) This was also demonstrated in the macroscopic appearance of the distal colon and (d) colon shortening (cm). Data represents >2 individual experiments (*n* = 5-8 mice). ^∗^*p* < 0.05, ^∗∗^*p* < 0.01, Villin^cre^IL-4R*α*^-/lox^ and IL-4R*α*^-/lox^ oxa vs. IL-4R*α*^-/lox^ etoh-control mice.

**Figure 2:**
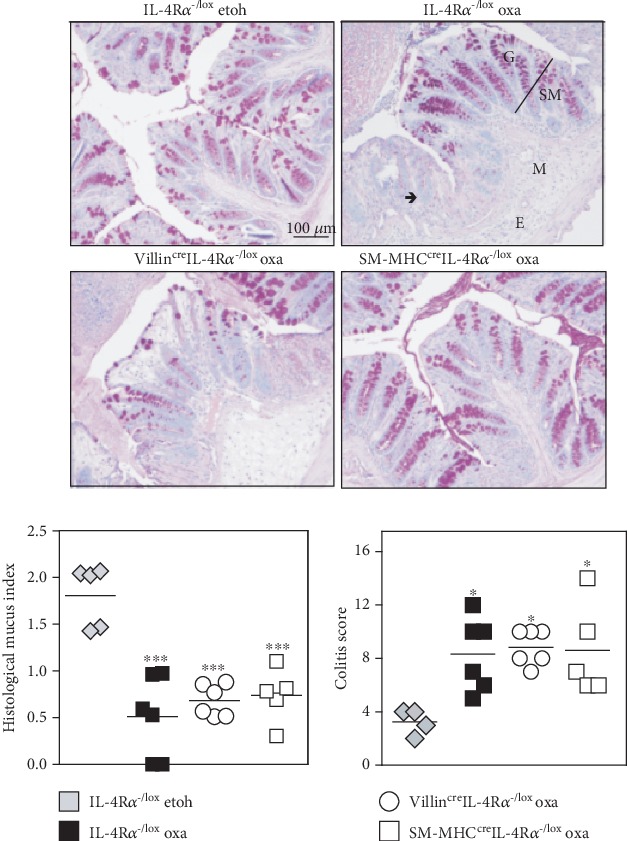
Mucus production in the colon of both epithelial and smooth muscle cell-specific IL-4R*α*-deficient mice. Distal colon sections from oxazolone-treated Villin^cre^IL-4R*α*^-/lox^ and SM-MHC^cre^IL-4R*α*^-/lox^ mice were stained with PAS, and mucus production was quantified and compared to IL-4R*α*^-/lox^ littermate controls treated with oxazolone or control mice treated with ethanol only. H&E-stained sections (see Figure 3) were scored for the presence of colitis with a maximum score of 15, and results were represented as the colitis score. SM = submucosa, M = mucosa, ➔ = infiltrating mononuclear cells, E = edema, and G = goblet cells. Data represents >2 individual experiments (*n* = 4-10 mice). ^∗^*p* < 0.05, Villin^cre^IL-4R*α*^-/lox^, SM-MHC^cre^IL-4R*α*^-/lox^, and IL-4R*α*^-/lox^ oxa vs. IL-4R*α*^-/lox^ etoh-control mice.

**Figure 3:**
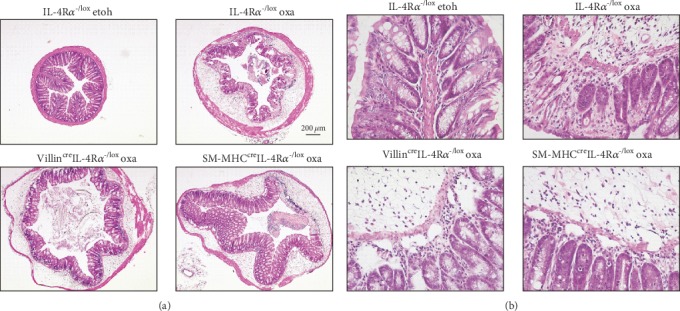
Histopathology of colitis in both epithelial and smooth muscle cell-specific IL-4R*α*-deficient mice. Distal colon sections from oxazolone-treated Villin^cre^IL-4R*α*^-/lox^ and SM-MHC^cre^IL-4R*α*^-/lox^ mice were stained with H&E to visualize inflammatory cell infiltration into the colon. Data represents 2 individual experiments (*n* = 4-10 mice).

Oxazolone induced a rapid onset colitis marked by weight loss (Figure 1(a)) in both hemizygous littermate controls (IL-4R*α*^-/lox^) and Villin^cre^IL-4R*α*^-/lox^ mice. This was accompanied by an increased distress score in both groups of mice (Figure 1(b)). Macroscopic examination revealed severe colitis limited to the distal half of the colon (Figure 1(c)), with inflammation-associated colon shortening (Figure 1(d)). Microscopic examination showed loss of mucus production (Figure 2) and superficial inflammation scored according to the presence of mononuclear cells, edema, infiltration of granulocytes, and epithelial layer disruption (Figure 3). To control for ethanol-induced inflammation [18], ethanol-only controls were included. While very mild inflammation and transient weight loss were induced in ethanol-only control mice, the disease parameters were significantly enhanced in oxazolone-treated mice. As hemizygous IL-4R*α*^-/lox^ littermate control mice have one allele deficient for IL-4R*α*, we wanted to confirm that this deficiency did not affect the disease outcome in any way. Comparing oxazolone-induced colitis between hemizygous IL-4R*α*^-/lox^ littermate and wild-type BALB/c mice, there was no significant difference in the colitis onset suggesting that a single IL-4R*α* allele is sufficient to mediate the disease onset. This was shown by comparable weight loss, distress score, and colon shortening (Supplementary Figure S1). These data suggest that inhibiting IL-4/IL-13 signalling specifically on small and large intestinal epithelial cells has no significant influence in the onset of colitis.

### 3.2. Oxazolone Colitis Develops in Smooth Muscle Cell-Specific IL-4R*α*-Deficient Mice

**Figure 4:**
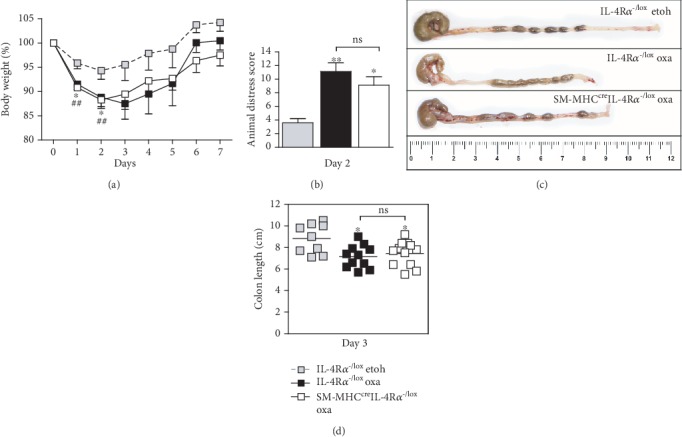
Oxazolone colitis develops in smooth muscle cell-specific IL-4R*α*-deficient mice. SM-MHC^cre^IL-4R*α*^-/lox^ mice were not protected from the onset of oxazolone-induced colitis compared with littermate control mice, shown by comparable (a) weight loss as a percentage of starting weight and (b) increased distress (day 2). (c) This was also demonstrated in the macroscopic appearance of the distal colon and (d) colon shortening (cm), data pooled from 2 experiments. Data represents >2 individual experiments, unless otherwise stated (*n* = 4-10 mice). ^∗^*p* < 0.05, ^∗∗^*p* < 0.01, SM-MHC^cre^IL-4R*α*^-/lox^ and IL-4R*α*^-/lox^ oxa vs. IL-4R*α*^-/lox^ etoh-control mice.

SM-MHC^cre^IL-4R*α*^-/lox^ mice treated with oxazolone presented with disease symptoms that appeared slightly reduced in some parameters such as animal distress and colitis score. However, this was not significantly reduced compared to hemizygous littermate controls; therefore, we concluded that SM-MHC^cre^IL-4R*α*^-/lox^ mice developed disease parameters consistent with colitis. Similar to epithelial cell-specific mice, this was shown by a rapid onset of disease marked by weight loss (Figure 4(a)) and an increased distress score in both groups of mice (Figure 4(b)). Macroscopic examination revealed severe colitis limited to the distal half of the colon (Figure 4(c)), with inflammation-associated colon shortening (Figure 4(d)). Lastly, microscopic examination showed loss of mucus production (Figure 2) and superficial inflammation scored according to the presence of mononuclear cells, edema, infiltration of granulocytes, and epithelial layer disruption (Figures 2 and 3). These data suggest that inhibiting IL-4/IL-13 signalling specifically on smooth muscle cells has no significant influence in the onset of colitis.

### 3.3. IL-4R*α* Signalling on Macrophages and Neutrophils Plays a Redundant Role in Oxazolone-Induced Colitis

**Figure 5:**
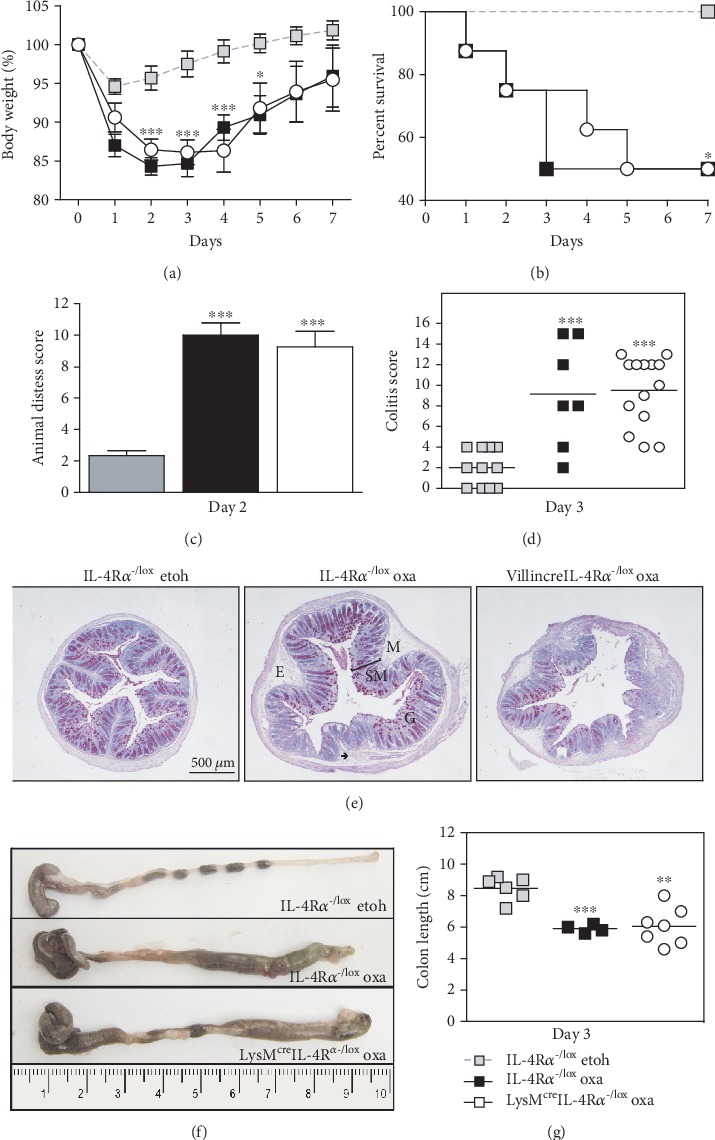
Oxazolone colitis develops in macrophage and neutrophil-specific IL-4R*α*-deficient mice. LysM^cre^IL-4R*α*^-/lox^ mice were not protected from the onset of oxazolone-induced colitis compared with littermate control mice, shown by comparable (a) weight loss as a percentage of starting weight, (b) survival, and (c) increased distress (day 2). (d) This was also demonstrated in the macroscopic appearance of the distal colon and (e) colon shortening (cm). Distal colon sections from oxazolone-treated LysM^cre^IL-4R*α*^-/lox^ mice were stained with PAS and scored for the presence of colitis and compared to wild-type littermate controls treated with oxazolone or control mice treated with ethanol only. Colitis was scored with a maximum score of 15, and results were represented as the colitis score. Data represents >2 individual experiments (*n* = 4-10 mice). ^∗∗^*p* < 0.01, ^∗∗∗^*p* < 0.001 vs. IL-4R*α*^-/lox^ etoh-only control mice. SM = submucosa, M = mucosa, ➔ = infiltrating mononuclear cells, E = edema, and G = goblet cells.

Oxazolone induced a rapid onset colitis marked by weight loss and mortality (Figures 5(a) and 5(b)) in both hemizygous littermate controls (IL-4R*α*^-/lox^) and LysM^cre^IL-4R*α*^-/lox^ mice. This was accompanied by an increased distress score in both groups of mice (Figure 5(c)). Microscopic examination showed superficial inflammation scored according to the presence of mononuclear cells, edema, infiltration of granulocytes, epithelial layer disruption, and loss of mucus production (Figures 5(d) and 5(e)). Macroscopic examination revealed severe colitis limited to the distal half of the colon (Figure 5(f)), with inflammation-associated colon shortening (Figure 5(g)). Furthermore, there seemed to be no difference in arginase and NOS2 activation in LysM^cre^IL-4R*α*^-/lox^ mice compared with littermate controls (Supplementary Figure S2). To control for ethanol-induced inflammation [18], ethanol-only controls were included.

**Figure 6:**
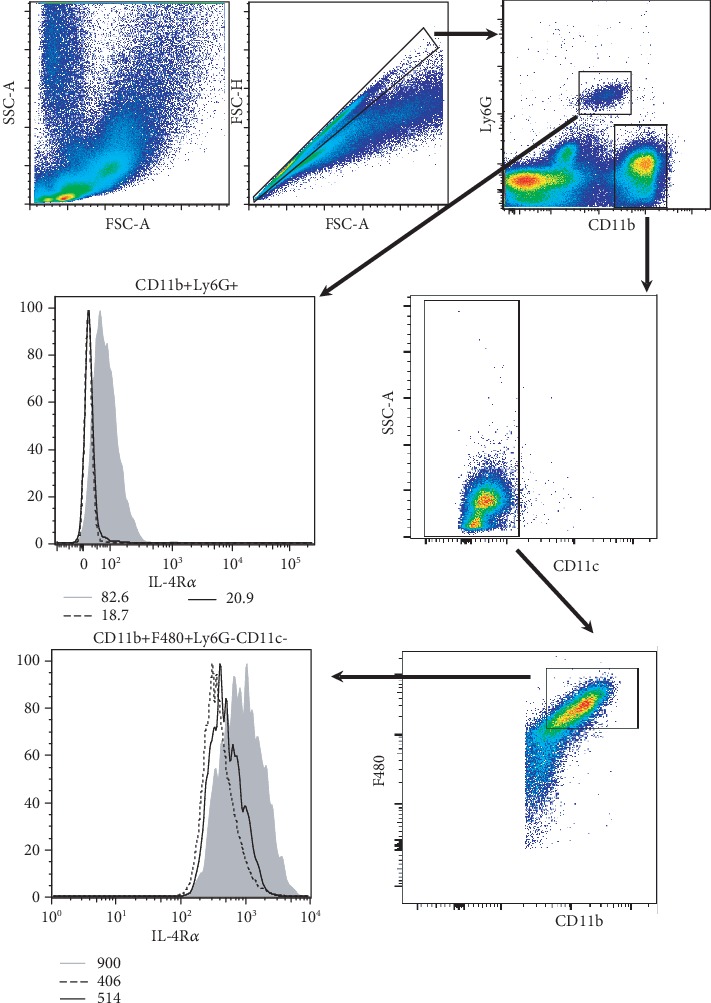
IL-4R*α* expression on macrophages and neutrophils in the peritoneal cavity. Single cells isolated by peritoneal lavage of naïve IL-4R*α*^-/lox^, IL-4R*α*^−/−^, and LysM^cre^IL-4R*α*^-/lox^ mice were stained for IL-4R*α* expression. Cells were gated on singlets, and dead cells were excluded. Macrophages were defined as CD11b+F4/80+CD11c-Ly6G-, and neutrophils were defined as CD11b+Ly6G+. Histograms represent 2-3 independent experiments (*n* = 3-4) with IL-4R*α*^-/lox^ = solid grey, IL-4R*α*^−/−^ = dashed line, and LysM^cre^IL-4R*α*^-/lox^ = black line.

We have previously characterized the efficiency of IL-4R*α* deletion on macrophages and neutrophils in the mesenteric lymph node of Schistosoma mansoni-infected mice [15] and lung [19]. To confirm the deletion of IL-4R*α* on cell populations associated with the colon, we used multicolor flow cytometry to analyze cells isolated from the peritoneal cavity of control IL-4R*α*^-/lox^, IL-4R*α*^−/−^, and LysM^cre^IL-4R*α*^-/lox^ mice. Cell populations known to express lysozyme M were gated as CD11b+F4/80+CD11c-Ly6G- “macrophages” and CD11b+Ly6G+ “neutrophils.” Neutrophils from IL-4R*α*^−/−^ and LysM^cre^IL-4R*α*^-/lox^ mice were completely deficient in IL-4R*α*, and macrophages were significantly depleted (Figure 6 and Supplementary Figure S3). The geometric mean from each strain is indicated in the corner of the histogram. Cells isolated from the lamina propria showed similar deletion of IL-4R*α* for CD11b+F4/80+CD11c-Ly6G- “macrophages” and CD11b+Ly6G+ “neutrophils,” but the expression was maintained on CD11b-F4/80-CD11c+Ly6G- “dendritic cells” on IL-4R*α*^-/lox^ and LysM^cre^IL-4R*α*^-/lox^ but not IL-4R*α*^−/−^ (Supplementary Figure S4). These data represent the results of 4 pooled colons per group. The repeat experiment provided similar results with geometric means as follows: (1) CD11b+F4/80+CD11c-Ly6G- “macrophages” IL‐4R*α*^‐/lox^ = 185, IL‐4R*α*^‐/‐^ = ‐136, and LysM^cre^IL‐4R*α*^‐/lox^ = ‐86.1; (2) CD11b+Ly6G+ “neutrophils” IL‐4R*α*^‐/lox^ = 99.5, IL‐4R*α*^‐/‐^ = 13.2, and LysM^cre^IL‐4R*α*^‐/lox^ = 7.06; and (3) CD11b-F4/80-CD11c+Ly6G- “dendritic cells” IL‐4R*α*^‐/lox^ = 72.2, IL‐4R*α*^‐/‐^ = 34.5, and LysM^cre^IL‐4R*α*^‐/lox^ = 65.8. Essentially, we found the efficient deletion of IL-4R*α* on neutrophils and a significant deletion on macrophages. Taken together, our data suggests that blocking IL-4/IL-13 signalling specifically on lysozyme M-positive macrophages and neutrophils has no significant influence in the onset of colitis.

## 4. Discussion

Oxazolone-induced colitis is a model for transient experimental colitis mediated by type 2 responses and resembles human ulcerative colitis. Our work described the induction of colitis in male BALB/c mice via intrarectal administration of oxazolone subsequent to skin sensitization [1–3, 7, 20]. Disease was characterized by a rapid onset inflammation peaking at day 2 postchallenge, which resulted in wasting disease and death or recovery. Histological assessment demonstrated a superficial colitis with ulceration and an inflammatory infiltrate of lymphocytes and granulocytes. To determine a role for IL-4/IL-13 responsive nonhematopoietic cells in either mediating the acute form of disease or limiting the disease severity, we used two different gene-deficient mouse strains. The first strain included mice selectively lacking the IL-4R*α* chain on intestinal epithelial cells (Villin^cre^IL-4R*α*^-/lox^) [8]. The second strain lacked the IL-4R*α* chain in smooth muscle cells (SM-MHC^cre^IL-4R*α*^-/lox^) [9, 10]. To establish a role of IL-4R*α* signalling on macrophages/neutrophils, we employed a mouse strain selectively lacking the IL-4R*α* chain on these cells (LysM^cre^IL-4R*α*^-/lox^) [15].

Here, we provide evidence that IL-4/IL-13 signalling on intestinal epithelial cells or smooth muscle cells is not important in mediating or preventing exacerbated acute oxazolone-induced colitis. As mentioned, the previously described effects of IL-13 on epithelial cells and the destruction of epithelial cells in ulcerative colitis provide a strong argument that perhaps IL-13 is contributing to disease pathology through epithelial cells. In addition to the effect IL-13 plays directly on epithelial cell function, IL-13 induces activation of the signal transducer and activator of transcription 6 (STAT6). Epithelial STAT6 has also been shown to be increased in pediatric subjects with ulcerative colitis [21]. It was also determined that epithelial cell apoptosis *in vitro* was STAT6-dependent and that STAT6 inhibition attenuated the IL-13-induced colon epithelial cell dysfunction [22]. Furthermore, STAT6 has previously been shown to alter epithelial barrier function and regulate Th2-inducing cytokine production [3]. Therefore, the epithelial cell-specific IL-4R*α*-deficient mouse strain would be able to directly test the effect of IL-13 signalling on epithelial cells and disease onset in oxazolone colitis.

Currently, there is very little known about the involvement of IL-4/IL-13 signalling on smooth muscle cells in ulcerative colitis. It has been shown that IL-4R*α*, IL-13R*α*1, and IL-13R*α*2 are expressed in the small intestine and colon of smooth muscle cells indicating a direct role of IL-4 and IL-13 on these cells [22]. Furthermore, IL-4/IL-13 signalling is associated with hypercontractility of smooth muscle cells, and treatment with IL-4/IL-13 *in vivo* causes an increase in contraction. This is most likely dependent on the STAT6 pathway [23, 24]. Hypercontraction is commonly described in helminth expulsion [25, 26], and as we have shown previously, smooth muscle cells that do not express IL-4R*α* have delayed *Nippostrongylus brasiliensis* clearance [9]. Interestingly, these mice also have a reduction in lung pathology associated with infection [27]. This was accompanied by reduced levels of IL-13 in these mice. We suggested that IL-4R*α*, smooth muscle cell-dependent cytokine production contributes to the initial recruitment of immune cells and initiating Th2 immunity to infection. Although it is clear that smooth muscle cells respond to IL-4/IL-13 in helminth infections, whether this influences the outcome in ulcerative colitis has not been studied. Although ulcerative colitis is described as an atypical Th2 response, patients demonstrate reduced smooth muscle contraction [28]. Hence, the smooth muscle cell-specific IL-4R*α*-deficient mouse strain provided us with a tool to understand the role of IL-4/IL-13-reponsive smooth muscle cells in oxazolone colitis. We hypothesized that the reduced Th2 pathology seen in SM-MHC^cre^IL-4R*α*^-/lox^ mice in response to helminths could be extrapolated to our oxazolone colitis model. However, we found only a very minor reduction in disease pathology and therefore concluded that IL-4R*α*-expressing smooth muscle cells play a redundant role in oxazolone colitis.

Previous studies have highlighted a role for the Th2 cytokines IL-4 and IL-13 in mediating acute ulcerative colitis [1, 5, 29, 30]. Furthermore, IL-13-producing NK T cells have been implicated as the main inducers of the associated disease pathology [3, 31, 32]. Our previous studies have also shown that both IL-4R*α*-responsive T cells and B cells play a role in driving the acute disease [2]. Considering that IL-4R*α*^−/−^ mice have an exacerbated disease phenotype [7], we set out to determine if other hematopoietic cell populations expressing IL-4R*α*, and prominent in the inflammatory response of ulcerative colitis, play a role in mediating disease. For this, we used LysM^cre^IL-4R*α*^-/lox^ BALB/c mice. This strain has been previously characterized by us [15, 19, 33]. Essentially, FACS analysis and subsequent functional studies revealed cell type-specific disruption of the IL-4R*α* gene in macrophages and neutrophils only for LysM^cre^IL-4R*α*^-/lox^ mice. Functions not affected by the deletion include IL-4/IL-13 responsiveness in T and B lymphocytes and dendritic cells, Th2/type 2 responses, and goblet cell hyperplasia, compared to wild-type littermate controls. This was confirmed in experimental helminth infections and allergy. There is conflicting data as to the role of M2 macrophages in the UC disease onset. Wild-type mice treated with the haptenating agent, oxazolone, develop UC and have an increase in the absolute number of F4/80+ colon macrophages and also pSTAT6+F4/80+ macrophages, suggesting an increase in alternative macrophage activation [21]. In contrast, IL-4-treated macrophages are able to attenuate oxazolone colitis [34]. In this study, oxazolone colitis was treatable with adoptively transferred IL-4-stimulated cryopreserved macrophages, likely alternatively activated macrophages. While it still remains to be determined if IL-4R*α* expression on macrophages plays a role in chronic oxazolone colitis, we conclude that IL-4R*α* expression on macrophages and neutrophils plays a redundant role in acute oxazolone colitis. Together, our data provides evidence that IL-4R*α* signalling on intestinal epithelial cells, smooth muscle cells, macrophages, and neutrophils is not essential in mediating or reducing the inflammatory responses that drive pathology in a mouse model of colitis.

## Figures and Tables

**Table 1:** Disease activity index scoring sheet (animal distress score).

Appearance	
Normal	0
General lack of grooming	1
Staring coat, ocular and nasal discharges	2
Piloerection, hunched up	3^∗^
Clinical signs	
Normal color and movement	0
Slight changes in activity	1
Moderate changes: weight loss, diarrhea	2
Severe changes: immobility, lameness	3^∗^
Natural behavior	
Normal	0
Minor changes	1
Less mobile and alert, isolated	2
Vocalization, restlessness, very still	3^∗^
Provoked behaviour	
Normal	0
Minor depression or exaggerated response	1
Moderate change in expected behaviour	2
Reacts violently or very weak	3^∗^

^∗^If 3 was scored more than once, an extra point was scored for every 3. *Total* = 16.

## Data Availability

The data used to support the findings of this study are included within the article and supplementary figures.

## Supplementary Materials

Supplementary Materials Supplementary Figure S1: BALB/c wild-type and IL-4R*α*^-/lox^ hemizygous littermate control mice develop comparable colitis. BALB/c wild-type and littermate control mice (IL-4R*α*^-/lox^) were treated with oxazolone and disease parameters compared, shown by (A) weight loss as a percentage of starting weight, (B) increased distress (day 2), and (C) colon shortening (cm). Data represents 2 individual experiments (*n* = 4-10 mice). ^∗^*p* < 0.05, ^∗∗^*p* < 0.01, and ^∗∗∗^*p* < 0.001 vs. IL-4R*α*^-/lox^ etoh-only control mice. ns = not significant, BALB/c oxa vs. IL-4R*α*^-/lox^ oxa. Supplementary Figure S2: oxazolone induces arginase and NOS2 production. LysM^cre^IL-4R*α*^-/lox^ mice were not protected from the onset of oxazolone-induced colitis despite a trend towards reduced arginase production, shown by PCR analysis of arginase production and NOS2 (A). This was also shown in histology sections stained specifically for arginase (B) and compared with etoh-only control mice. Data represents 2 individual experiments (*n* = 4-10 mice). ns = not significant. Supplementary Figure S3: IL-4R*α* expression on peritoneal macrophages and neutrophils. Single cells isolated by peritoneal gavage of naïve IL-4R*α*^-/lox^, IL-4R*α*^−/−^, and LysM^cre^IL-4R*α*^-/lox^ mice were stained for IL-4R*α* expression. Cells were gated on singlets, and dead cells were excluded. Macrophages were defined as CD11b+F4/80+CD11c-Ly6G-, and neutrophils were defined as CD11b+Ly6G+. Bar graphs of mean fluorescent intensity of IL-4R*α* expression on macrophages (A) or neutrophils (B) represents 2 independent experiments (*n* = 3-4). Supplementary Figure S4: IL-4R*α* expression on lamina propria cell populations. Single cells isolated from the lamina propria of naïve IL-4R*α*^-/lox^, IL-4R*α*^−/−^, and LysM^cre^IL-4R*α*^-/lox^ mice were stained for IL-4R*α* expression. Cells were gated on singlets, and dead cells were excluded. Macrophages were defined as CD11b+F4/80+CD11c-Ly6G-, and neutrophils were defined as CD11b+Ly6G+. Dendritic cells were defined as CD11b-F4/80-CD11c+Ly6G-. Histograms represent 2 independent experiments (*n* = 4 pooled colons) with IL-4R*α*^-/lox^ = solid grey, IL-4R*α*^−/−^ = dashed line, and LysM^cre^IL-4R*α*^-/lox^ = black line.
