# Supplementary material for: IL-4 Receptor-Alpha Signalling of Intestinal Epithelial Cells, Smooth Muscle Cells, and Macrophages Plays a Redundant Role in Oxazolone Colitis
Source: Mediators Inflamm. 2020 Jan 17;2020:4361043. doi: 10.1155/2020/4361043 (PMC7201672; doi:10.1155/2020/4361043)
Supplement: Supplementary Materials — Supplementary Figure S1: BALB/c wild-type and IL-4Rα-/lox hemizygous littermate control mice develop comparable colitis. BALB/c wild-type and littermate control mice (IL-4Rα-/lox) were treated with oxazolone and disease parameters compared, shown by (A) weight loss as a percentage of starting weight, (B) increased distress (day 2), and (C) colon shortening (cm). Data represents 2 individual experiments (n = 4-10 mice). ∗p < 0.05, ∗∗p < 0.01, and ∗∗∗p < 0.001 vs. IL-4Rα-/lox etoh-only control mice. ns = not significant, BALB/c oxa vs. IL-4Rα-/lox oxa. Supplementary Figure S2: oxazolone induces arginase and NOS2 production. LysMcreIL-4Rα-/lox mice were not protected from the onset of oxazolone-induced colitis despite a trend towards reduced arginase production, shown by PCR analysis of arginase production and NOS2 (A). This was also shown in histology sections stained specifically for arginase (B) and compared with etoh-only control mice. Data represents 2 individual experiments (n = 4-10 mice). ns = not significant. Supplementary Figure S3: IL-4Rα expression on peritoneal macrophages and neutrophils. Single cells isolated by peritoneal gavage of naïve IL-4Rα-/lox, IL-4Rα−/−, and LysMcreIL-4Rα-/lox mice were stained for IL-4Rα expression. Cells were gated on singlets, and dead cells were excluded. Macrophages were defined as CD11b+F4/80+CD11c-Ly6G-, and neutrophils were defined as CD11b+Ly6G+. Bar graphs of mean fluorescent intensity of IL-4Rα expression on macrophages (A) or neutrophils (B) represents 2 independent experiments (n = 3-4). Supplementary Figure S4: IL-4Rα expression on lamina propria cell populations. Single cells isolated from the lamina propria of naïve IL-4Rα-/lox, IL-4Rα−/−, and LysMcreIL-4Rα-/lox mice were stained for IL-4Rα expression. Cells were gated on singlets, and dead cells were excluded. Macrophages were defined as CD11b+F4/80+CD11c-Ly6G-, and neutrophils were defined as CD11b+Ly6G+. Dendritic cells were defined as CD11b-F4/80-CD11c+Ly6G-. [file 4361043.f1.pdf]

Supplementary Materials and Methods

Quantitative PCR

Colon samples (0.5 cm-length) were snap frozen in Qiazol (Qiagen, Germany) and stored at -80C. The samples were homogenized in Qiazol to ensure efficient lysis of the tissue and total RNA isolated from the lysate using RNeasy Mini kit according to the manufacturer's instructions. RNA quantity and purity were measured using the ND-1000 NanoDrop spectrophotometer (ThermoScientific, DE, USA). For gene expression analysis, 500 ng total RNA was reverse-transcribed into cDNA using Transcriptor First Strand cDNA Synthesis Kit (Roche, Germany) according to the manufacturer's instructions. Quantitative real-time PCR (qPCR) was performed using LightCycler® 480 SYBR Green I Master (Roche, Germany) and gene-specific primers (IDT, CA, USA) for Nos2 (Forward: 5'-AACTGCAAGAGAACGGAGAACG-3', Reverse: AACATTCTGTGCTGTCCCAGT-3'), Arg1 (Forward: 5'-CAGAAGAATGGAAGAGTCAG-3', Reverse: 5'-CAGATATGCAGGGAGTCACC-3') and Hprt (Forward: 5'-GTTGGATATGCCCTTGAC-3', Reverse: 5'-AGGACTAGAACACCTGCT-3'). Absolute quantification for each primer was performed according to LightCycler® 480 Software and gene expression levels were normalized to Hprt housekeeping gene.

Supplementary Figure 1

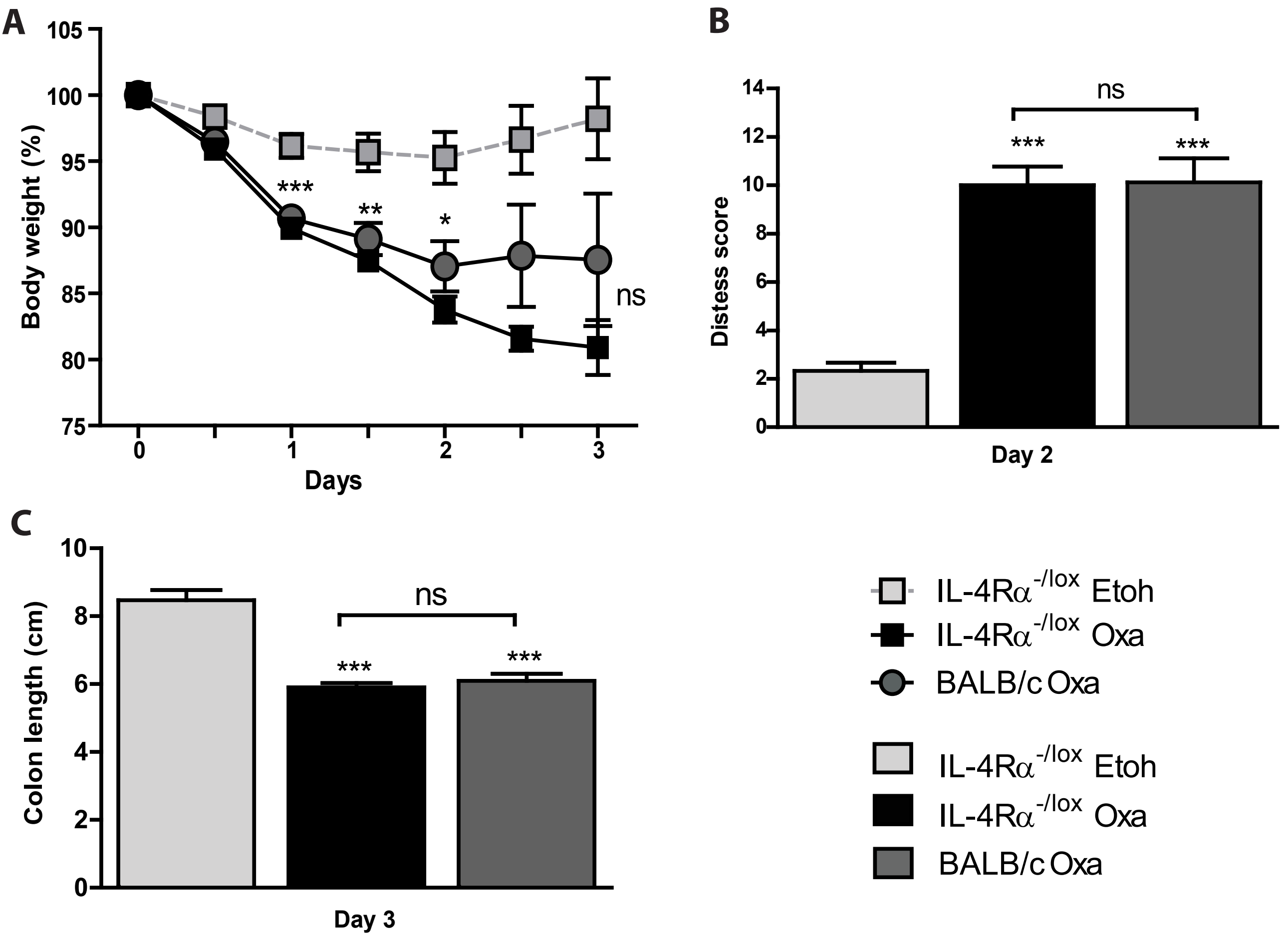

Supplementary Figure S1. BALB/c wild-type and IL-4R$^{-/lox}$ hemizygous littermate control mice develop comparable colitis. BALB/c wild-type and littermate control mice (IL-4Rα$^{-/lox}$) were treated with oxazolone and disease parameters compared, shown by (A) weight loss as a percentage of starting weight, (B) increased distress (day2) and (C) colon shortening (cm). Data represents 2 individual experiments (n=4-10 mice) and *=p<0.05, **=p<0.01 and ***=p<0.001 vs. IL-4Rα$^{-/lox}$ etoh-only control mice, ns=not significant, BALB/c oxa vs. IL-4Rα$^{-/lox}$ oxa.

Supplementary Figure 2

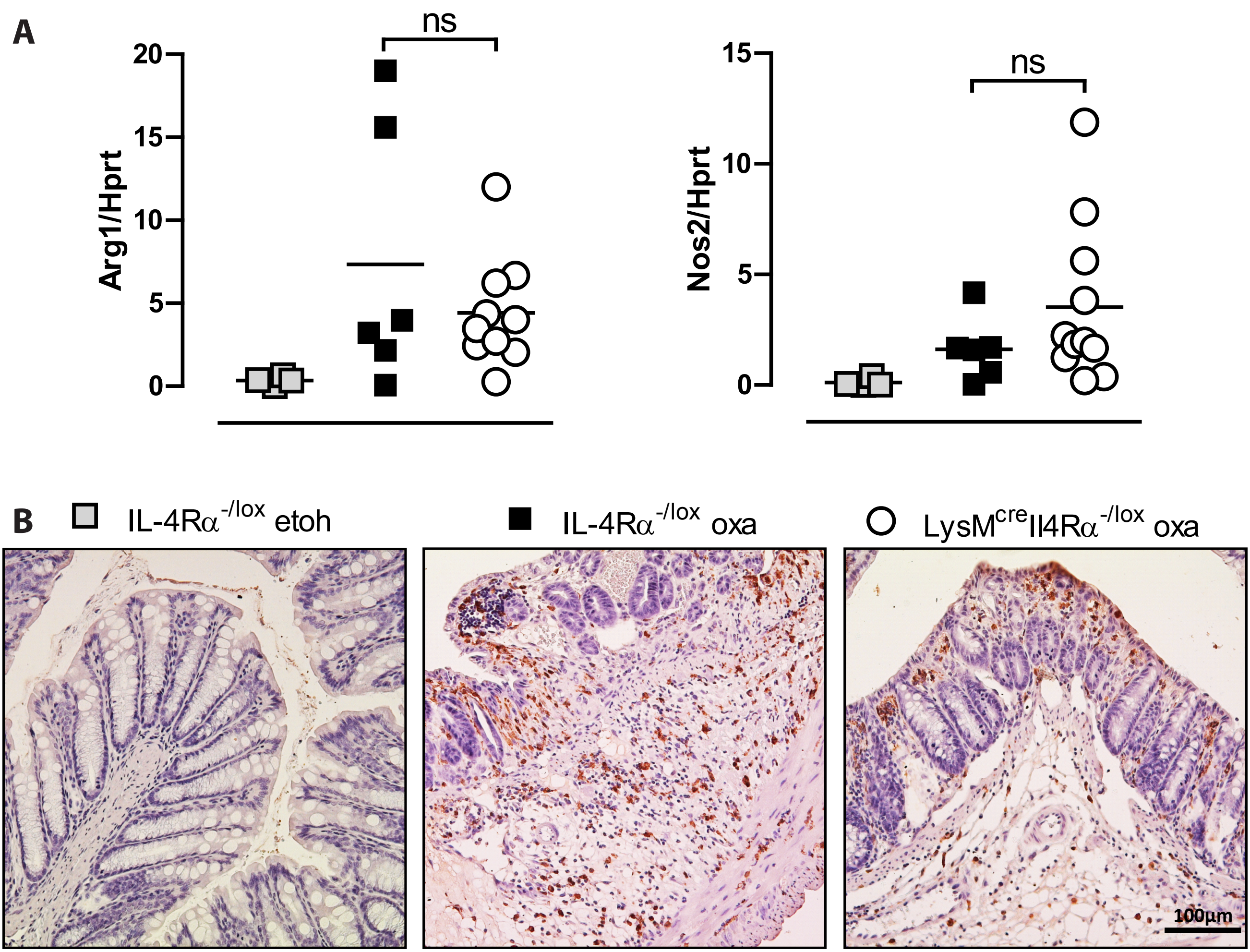

Supplementary Figure S2. Oxazolone induces arginase and NOS2 production. LysM^{cre}IL-4Rα$^{-/lox}$ mice were not protected from the onset of oxazolone-induced colitis despite a trend towards reduced arginase production. Shown by PCR analysis of arginase production and NOS2 (A). This was also shown in histology sections stained specifically for arginase (B) and compared with etoh only control mice. Data represents 2 individual experiments (n=4-10 mice) and ns= not significant.

Supplementary Figure 3

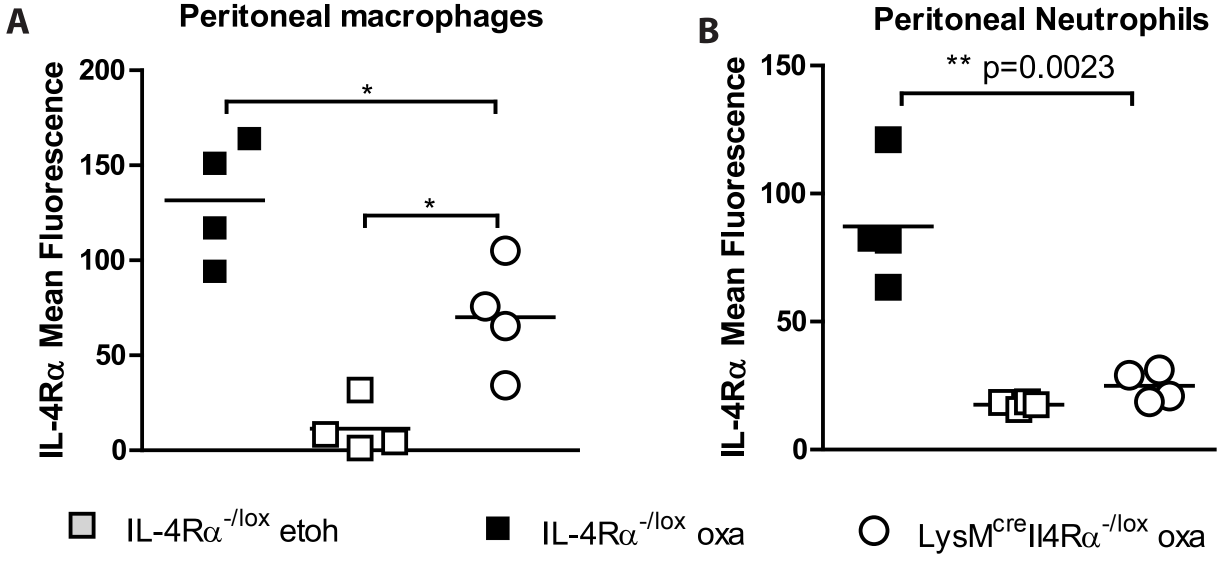

Supplementary Figure 3: IL-4Rα expression on peritoneal Macrophages and Neutrophils. Single cells isolated by peritoneal gavage of naïve IL-4Rα$^{-/lox}$, IL-4Rα$^{-/-}$ and LysM^{cre}IL-4Rα$^{-/lox}$ mice were stained for IL-4Rα expression. Cells were gated on singlets and dead cells were excluded. Macrophages were defined as CD11b+F4/80+CD11c-Ly6G- and neutrophils were defined as CD11b+Ly6G+. Bar graphs of Mean Fluorescent Intensity of IL-4Rα expression on Macrophages (A) or Neutrophils (B) represents 2 independent experiments (n= 3-4).

Supplementary Figure 4

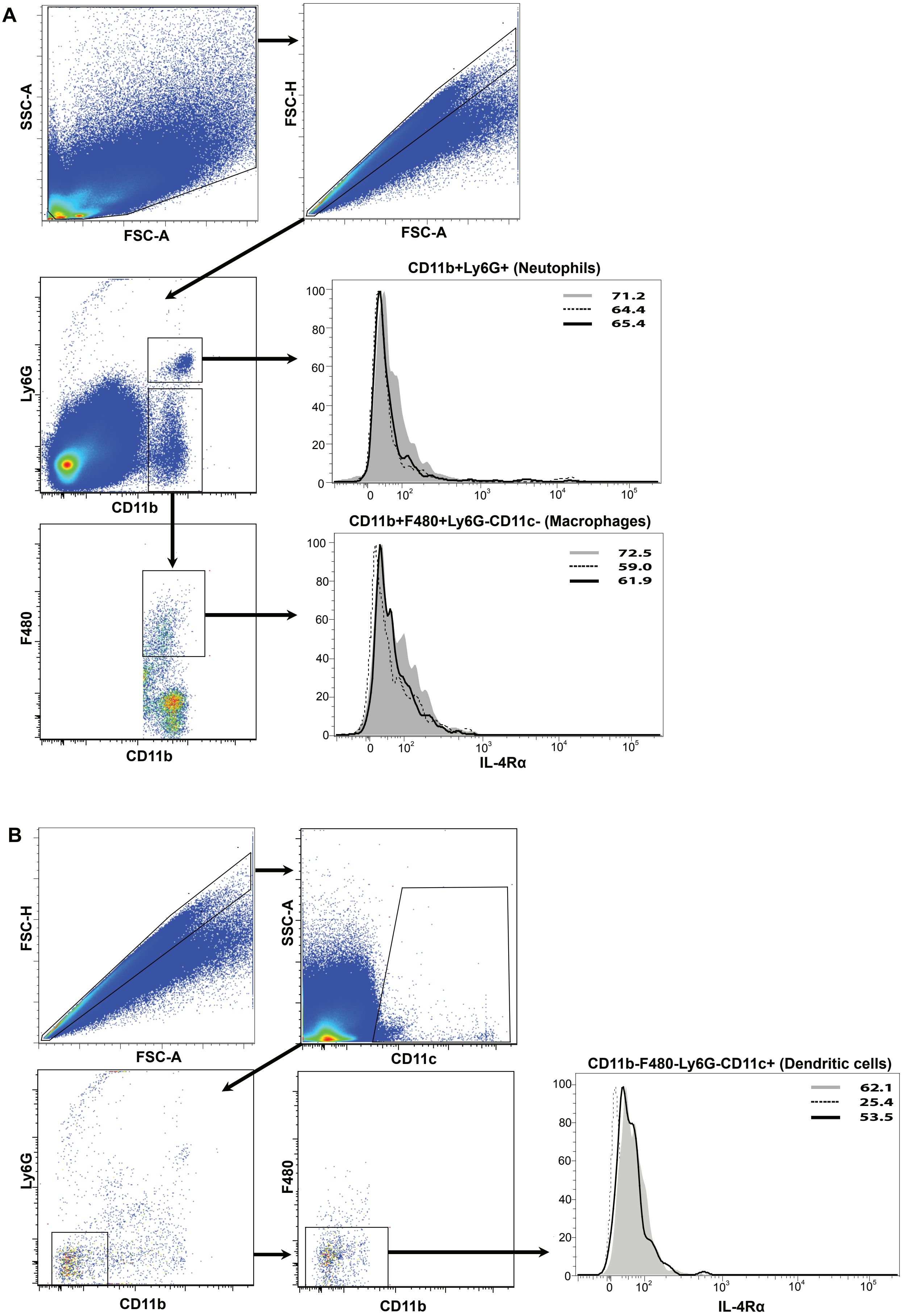

Supplementary Figure S4. IL-4Rα expression on lamina propria cell populations. Single cells isolated from the lamina propria of naïve IL-4Rα$^{-/lox}$, IL-4Rα$^{-/-}$ and LysM^{cre}IL-4Rα$^{-/lox}$ mice were stained for IL-4Rα expression. Cells were gated on singlets and dead cells were excluded. Macrophages were defined as CD11b+F4/80+CD11c-Ly6G- neutrophils were defined as CD11b+Ly6G+. Dendritic cells were defined as CD11b-F4/80-CD11c+Ly6G-. Histograms represent 2 independent experiments (n= 4 pooled colons) with IL-4Rα$^{-/lox}$ = solid grey, IL-4Rα$^{-/-}$ = dashed line and LysM^{cre}IL-4Rα$^{-/lox}$ = black line.
